# Short cell cycle duration is a phenotype of human epidermal stem cells

**DOI:** 10.1186/s13287-024-03670-y

**Published:** 2024-03-13

**Authors:** Tong Xiao, Ugomma C Eze, Alex Charruyer-Reinwald, Tracy Weisenberger, Ayman Khalifa, Brook Abegaze, Gabrielle K Schwab, Rasha H Elsabagh, T. Richard Parenteau, Karl Kochanowski, Merisa Piper, Yumin Xia, Jeffrey B Cheng, Raymond J Cho, Ruby Ghadially

**Affiliations:** 1 Department of Dermatology, The Second Affiliated Hospital of Xi’an Jiaotong University, Xi’an, Shaanxi China; 2 Department of Dermatology, San Francisco Co-Director Epithelial Section Eli and Edythe Broad Center of Regeneration Medicine and Stem Cell Research, University of California, 1700 Owens Street, San Francisco, CA 94158 USA; 3 Department of Dermatology, VA Medical Center, San Francisco, CA USA; 4 Faculty of Science, Zagazig University, Zagazig, Egypt; 5 Immunology Department, Animal Health Research Institute (AHRI), Giza, Egypt; 6 Division of Geriatrics, UC San Francisco, San Francisco, CA USA; 7 Department of Pharmaceutical Chemistry, UC San Francisco, San Francisco, CA USA; 8 Department of Plastic Surgery, UC San Francisco, San Francisco, CA USA

**Keywords:** Keratinocyte, Stem cell, Progenitor, Cell cycle duration, Live cell imaging, Single-cell RNA sequencing, Cell cycle progression, Differentiation, Time-lapse microscopy, Cell tracking

## Abstract

**Background:**

A traditional view is that stem cells (SCs) divide slowly. Meanwhile, both embryonic and pluripotent SCs display a shorter cell cycle duration (CCD) in comparison to more committed progenitors (CPs).

**Methods:**

We examined the in vitro proliferation and cycling behavior of somatic adult human cells using live cell imaging of passage zero keratinocytes and single-cell RNA sequencing.

**Results:**

We found two populations of keratinocytes: those with short CCD and protracted near exponential growth, and those with long CCD and terminal differentiation. Applying the ergodic principle, the comparative numbers of cycling cells in S phase in an enriched population of SCs confirmed a shorter CCD than CPs. Further, analysis of single-cell RNA sequencing of cycling adult human keratinocyte SCs and CPs indicated a shortening of both G1 and G2M phases in the SC.

**Conclusions:**

Contrary to the pervasive paradigm, SCs progress through cell cycle more quickly than more differentiated dividing CPs. Thus, somatic human adult keratinocyte SCs may divide infrequently, but divide rapidly when they divide. Additionally, it was found that SC-like proliferation persisted in vitro.

**Supplementary Information:**

The online version contains supplementary material available at 10.1186/s13287-024-03670-y.

## Background

Epidermal homeostasis is proposed to be maintained by stem cells (SC) that proliferate for the long-term and both self-renew and produce committed progenitor (CP) /transit amplifying cells that undergo limited numbers of divisions before terminal differentiation [1–3]. An alternative paradigm proposes a stochastic model where all dividing keratinocytes are functionally equivalent and generate dividing and differentiating daughters with equal probability [4, 5].

Notably, with the advent of human keratinocyte cell culture, Barrandon and Green found three types of proliferative keratinocytes; holoclones, meroclones, and paraclones [6]. Holoclones produced large proliferative colonies, paraclones produced terminally differentiating colonies, and meroclones were transitional between holoclones and paraclones. More recently, live cell imaging of individual keratinocytes revealed only two types of cells, those that continuously divide and form expanding colonies and those that gradually arrest the cell cycle [7]. The expectation of a stochastic process based on a single progenitor would be a range of colony size, however, Nanba found a specific phenotype that was associated with continuously expanding/ SC colonies versus differentiating colonies.

SCs are believed to divide slowly [8], implying a longer cell cycle duration (CCD). However, periods of inactivity in G0 (infrequent cycling) would produce some of the same effects as a longer CCD. Recent studies of embryonic SCs have challenged the paradigm of a slowly dividing SC. For example, in human embryonic SCs, G1 is shortened [9] and lengthened G1 indicates differentiation [10]. In murine pluripotent SCs, G1 and G2 phases are also truncated [11, 12]. While information on somatic cells is limited, murine adult neural SCs were shown to increase time in both G1 and G2 phases as they differentiated [13]. Thus, despite the early-held belief that SCs divide slowly, recent studies that produce a more granular understanding of SC proliferation challenge this notion.

Traditionally, CCD has been evaluated using the ergodicity assumption; that is, within asynchronous populations at a single timepoint, the proportion of cells found to be in a given cell cycle phase is equal to the proportion of time a single cell spends in that phase relative to the total CCD [14–16]. Commonly, to determine the proportion of cells in S phase, markers such as EdU or BrdU are used. If the CCD of one population of cells is longer than that of another, a smaller proportion of cells will be in S phase, assuming that modifications of CCD result from changes in G1 and/or G2M, not S [17–21].

However, when cells can be imaged and tracked longitudinally, CCD is most easily measured based on the interval between two consecutive mitoses, as cell division is the most morphologically recognizable event in the cell cycle [22].

Finally, single-cell RNA sequencing has provided a new avenue to assess CCD. Data acquired from asynchronous samples can be synchronized during data analysis, followed by reconstruction of cell cycle phase from gene expression data [23–27]. This method allows determination of the phase responsible for changes in CCD observed.

Embryonic and pluripotent SCs cycle more quickly than the differentiated progenitors they produce. Still, it is unclear whether these cells cycle more quickly in relation to their innate stemness, or instead in relation to their pluripotency. Here we study the CCD of somatic SCs, which are committed to a defined lineage. Here we use 3 complementary methods to show that adult keratinocyte SCs have a shorter CCD than their differentiated progenitor daughters. We first used live cell imaging to examine the proliferation and cycling behavior of individual freshly-isolated passage 0 normal adult human keratinocytes, in vitro, for up to 16 days. We then compared the CCD of SCs and more differentiated CPs using live cell imaging, the proportions of S phase labeled keratinocytes in vitro, and single-cell RNA sequencing.

## Materials and methods

### Isolation of human keratinocytes

Human skin surgical discards were obtained with appropriate approvals from the UCSF Committee on Human Research and VA Medical Center, San Francisco. All studies abided by the rules of the Internal Review Board and the tenets of the Declaration of Helsinki. Keratinocytes were isolated from human skin surgical discard samples. Skin samples were from individuals 34, 36, and 49 years old (live cell imaging), and 32, 42, 39, 68, and 70 years (immunofluorescence). Samples were placed in Dispase 25U/ml (Gibco) overnight, followed by peeling of epidermis. 0.05% trypsin-EDTA (Gibco) and 10% TNS (Gibco) were then used to dissociate single cells. All cells were placed at 37ºC with 5% CO_2_ in 154CF medium (Gibco, live cell imaging) or CnT-PR medium (CELLnTEC, immunofluorescence).

### Live cell imaging

For time-lapse imaging, cells were plated on Incucyte® ImageLock 96-well plates (Essen Biosciences, Cat #4379), sealed with BreathEasy foil to prevent evaporation (Neta Scientific, Cat# RPI-248,738). Keratinocytes were plated at 90,000-120,000 cells/cm^2^ in 96-well plates. Approximately 5% of cells attached. Culture media was replaced after 24 h, at which time cells were again lost, providing analysis of approximately 50–200 cells per 1.95mm^2^ field and resulting in 0–3 colonies per field. Thereafter, medium was replaced every 2–3 days without disturbing the cells and the culture dish was placed in the same location in the microscope to be able to follow the same field of view. Cells were imaged every 20 min for 11–16 days using a Incucyte® S3 automated imaging system (Essen BioScience) using its 10X objective and 1–3 predefined fields of view.

### Lineage analysis

Lineage trees were generated by manual analysis of time lapse photography. Colonies were identified in the final frame and were first viewed backwards to ensure the colony remained in field, and then forwards from the first division of the founder cell, noting each cell division on an Excel spreadsheet. For large colonies it often proved infeasible to track all divisions. Cell cycle duration was recorded for the second and subsequent divisions.

### Immunostaining/ S phase labeling

Keratinocytes were isolated and plated at 140,000 cells/cm^2^ in 8-well chambers overnight, before immunofluorescence staining (Invitrogen). Keratinocytes were incubated with EdU for 1 hour, followed by Click-iT®-555 detection. Cells were then fixed with 4% paraformaldehyde, then incubated with anti-ki67 (AB_11000602) and anti-cytokeratin 10 (AB_629835) primary antibodies, followed by Alexa Fluor 488 (AB_2535792) and Cy5 (AB_955068) secondary antibodies. 4’,6-diamidino2-phenylindole (DAPI, 100 mg/ml) was used to identify nuclei. Cells were photographed using an DM4B fluorescence microscope (Leica) and separate channels were merged and analyzed by LAX and ImageJ. A small percent of the K10(-) cells could be melanocytes (5–10% basal keratinocytes) or Langerhans cells (1.8%). However, only few percent of melanocytes in the basal layer are Ki67 + so that the effect on our results would be minimal.

### Single-cell RNA sequencing and cell cycle phase analysis

The raw dataset of human epidermis from Cheng et al. 2018 is available in the European Genome-Phenome Archive (EGA) under accession number EGAS00001002927. We selected samples from normal trunk (*n* = 3). Cells with fewer than 500 genes per cell and cells with greater than 10% mitochondrial content were discarded. Batch effects (by sample) were removed by normalizing all cells in a batch to the most counts, and then multiplying by the median counts in that batch. These normalized counts were merged together across samples and log2 normalization was performed. Using default parameters of Seurat v.4.3.0, variable genes were identified, and the counts were scaled by regressing out each batch. Principal component analysis (PCA) was performed, and the significant PCs, which were identified using the JackStraw function, were carried forward for analysis. The nearest neighbors and clustering was performed using default Seurat v4.3.0 parameters.

Cluster markers were interpreted and assigned cluster identity by using canonical basal keratinocyte annotations, such as *KRT5, KRT14, ITGA6* and *ITGB1*. Subclustering within the mitotic group was performed by selecting and clustering the cells identified by expression of *MKI67*, and repeating the clustering procedure with batch effect correction. SCs were identified based on the relatively high expression of genes described from Enzo et al. corresponding to the holoclone signature. CPs were identified based on the relative low expression of the holoclone signature, corresponding to the meroclone and paraclone groups [28].

Using the tricycle analysis tool in Seurat v4.3.0, cell cycle phase scores were calculated based on canonical G2M and S phase markers, and the putative cell cycle phase (G1, G2/M or S) was assigned to each cell. The proportion of cells in each phase was reported for holoclone, meroclone and paraclone groups.

### Statistical analysis

All data were analyzed in GraphPad prism 9.0. Ordinary one-way ANOVA and Kruskal-Wallis test were used to test mean or median difference between multiple groups, respectively. Unpaired Student’s *t* test, paired *t* test, and Mann-Whitney U test were used to compare the means or medians between two groups. Significance was defined as *P* < 0.05. Statistical details of experiments can be found in the Results section and Figure legends.

## Results

### Primary adult human keratinocytes form colonies that either continue near-exponential expansion or terminally differentiate

**Fig. 1:**
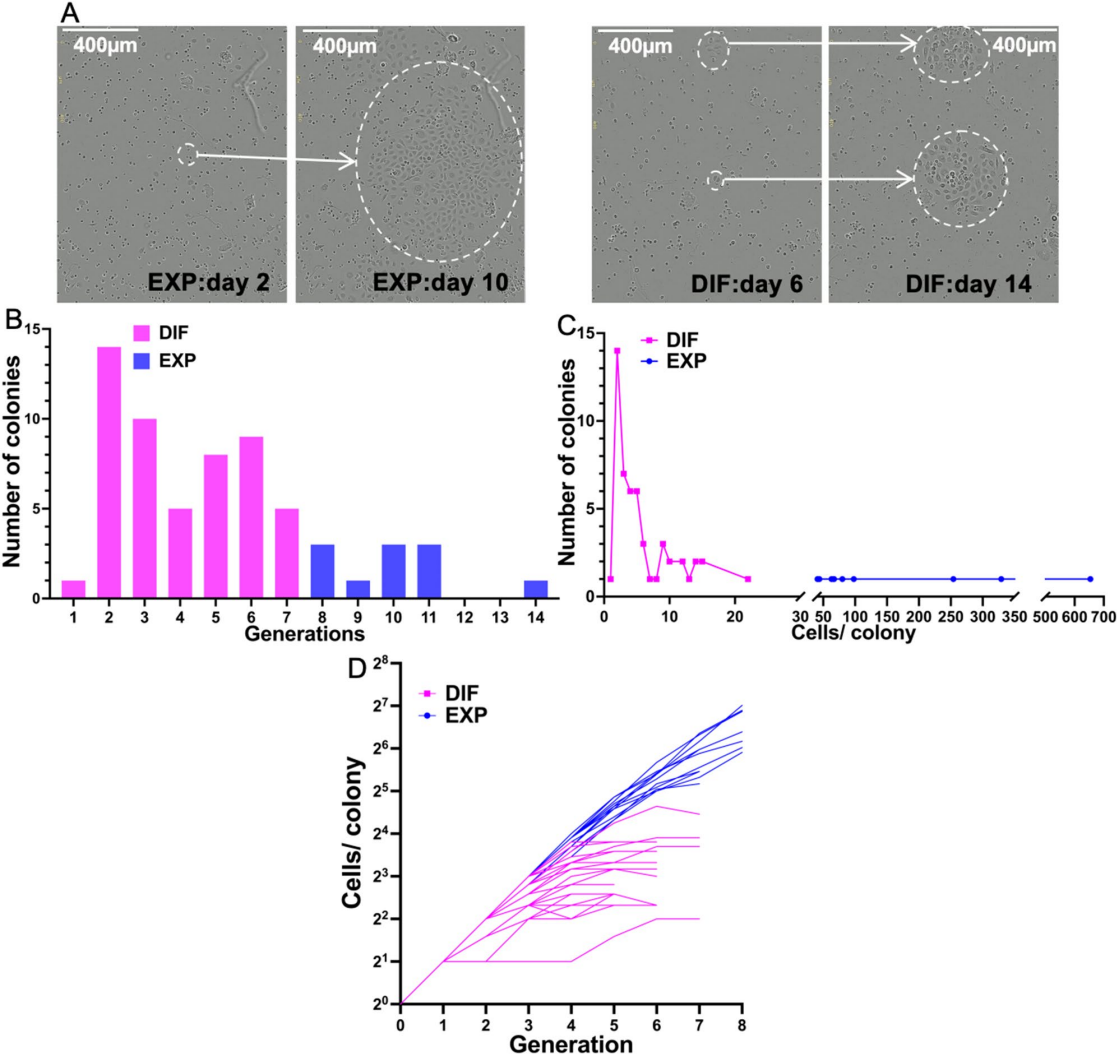
In vitro, individual adult human keratinocytes produce colonies that either continue to expand exponentially or undergo terminal differentiation. (**A**) Sample expanding and differentiating colonies at near the beginning and end of imaging. Scale bar 400 μm. (**B**) Total number of generations before terminal differentiation, or till end of observation. (**C**) Number of colonies of different sizes for expanding and differentiating colonies. (**D**) Plot of cell number over generations, for expanding and differentiating colonies. EXP: expanding colonies. DIF: differentiating colonies

Time lapse imaging of freshly-isolated passage 0 adult human keratinocytes from 3 donors (34, 36, and 42 years old) was performed for 11–16 days and images were recorded every 20 min [Fig. 1A, Additional file 1: Video S1 (differentiating colony) and Additional file 2: Video S2 (expanding colony)]. Keratinocytes that were imaged while growing showed equivalent colony formation to non-imaged controls. Since more differentiated cells are rapidly lost during cell culture, and to avoid cell damage, we did not enrich for SCs and progenitors by cell sorting. Furthermore, sample size made this infeasible.

The videos from 3 donors revealed a total of 65 colonies that could be analyzed. We constructed lineage trees for these 65 colonies, including 1191 cell cycles (Additional file 3: Spreadsheet S1, Additional file 4: Fig. S1). Each colony was tracked from the first division onwards.

In keeping with previous studies [7, 29], we found that more than 97% of divisions occurred within 48 h of the previous division, if a further division was to occur. Consequently, cells not dividing within 48 h were categorized as differentiated (D) and those that divided as proliferative (P). Cells that could not be tracked for 48 h were classified as unknown (U) and excluded from further analysis. For large colonies it often proved infeasible to track all divisions as the colony grew large and tended to end up offscreen.

Live cell imaging revealed two types of colonies; those that continued expanding (13 colonies, 20%) and those that terminally differentiated (52 colonies, 80%) during the observation period (Fig. 1A). Expanding colonies exhibited 8–14 generations during the observation period, whereas differentiating colonies underwent 1–7 generations (10 ± 0.5 vs. 4 ± 0.2, *P* < 0.0001, *t* test) (Fig. 1B).

This resulted in the mean final colony size of differentiating colonies being significantly smaller than that of expanding colonies at termination of imaging (158 ± 57.2 vs. 5.8 ± 0.6, *P* < 0.0001, *t* test) (Fig. 1C). The expanding colonies increased in cell number almost exponentially throughout the observation period, while differentiating colonies increased more slowly and gradually ceased cell division (Fig. 1D). Here we show cell counts of expanding colonies within 8 generations, where the majority of colonies were still traceable, since most of the expanding colonies went significantly off screen before termination of the videos.

In summary, differentiated colonies were those that terminally differentiated (cells all became D), with no cell divisions for at least 48 h before the end of the video. In contrast, expanding colonies were those that continued to divide almost exponentially (Fig. 1D) during the entire observation period.

### While expanding colonies maintain predominantly proliferative divisions, differentiating colonies undergo progressively more differentiation divisions with each generation

**Fig. 2:**
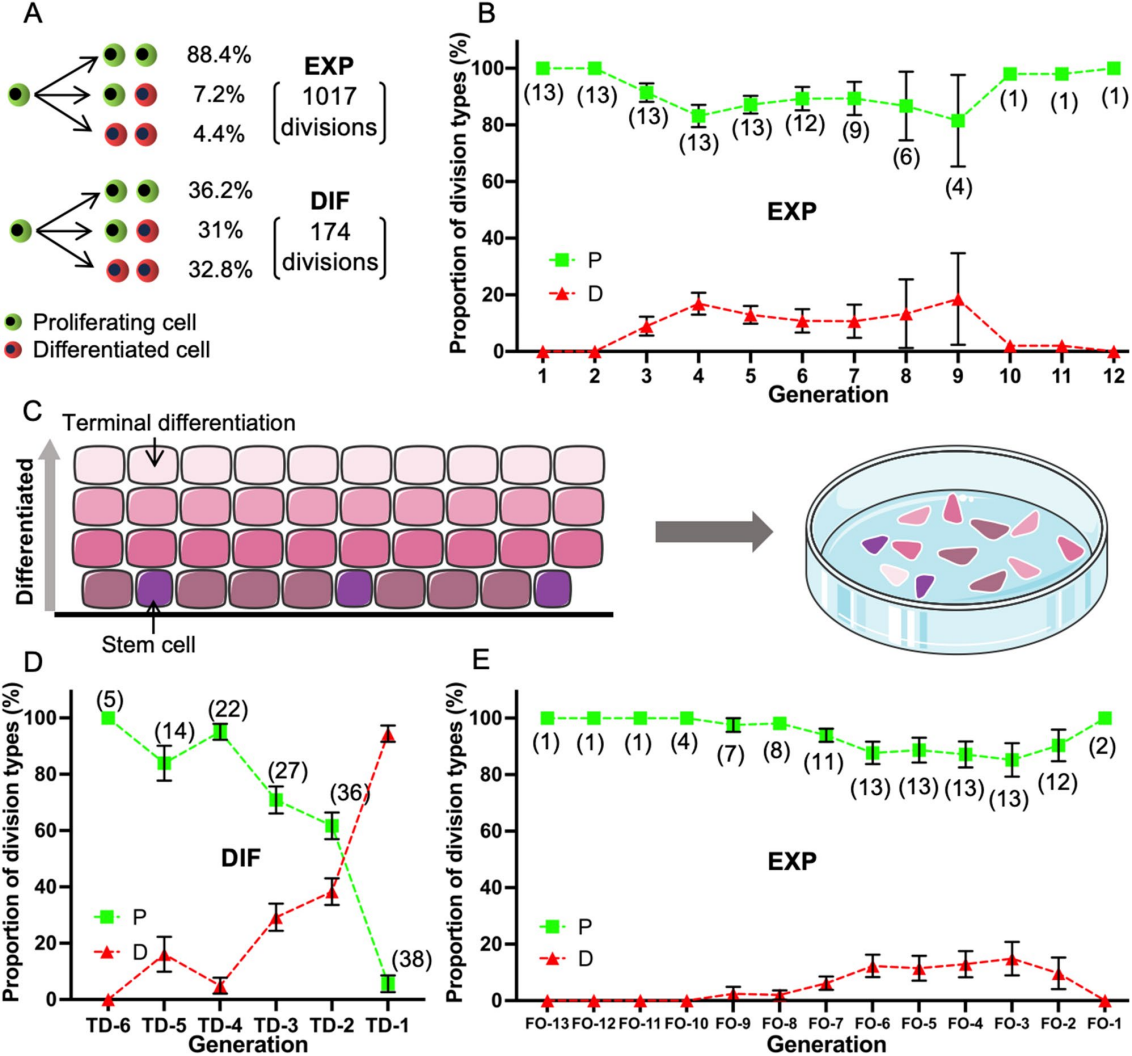
Expanding colonies show predominantly PP divisions over 12 generations in vitro, while differentiating colonies show an increased likelihood of DD divisions at subsequent generations. (**A**) Proportion of division types (PP, PD, DD) in expanding and differentiating colonies. (**B**) Mean proportion of P and D cells produced in expanding colonies over generations in vitro. One-way ANOVA, *P* = 0.09. (**C**) Plating freshly obtained cells results in keratinocytes with a range of differentiation status. (**D**) Mean proportion of P and D cells produced in differentiating colonies, examined from the terminal differentiation division backwards. One-way ANOVA, *P* < 0.0001. (**E**) Mean proportion of P and D cells produced in expanding colonies over time, examined from the final observed division backwards. One-way ANOVA, *P* = 0.57. Data presented as mean ± SEM. (): colonies analyzed. TD: terminal differentiation. FO: final observation. EXP: expanding colonies. DIF: differentiating colonies

For each cell division, three outcomes are possible: (1) both daughter cells proliferate (PP); (2) one daughter proliferates but the other differentiates (PD); or (3) both daughter cells differentiate (DD). Using live cell imaging from the same 3 adult donors, for divisions observed in the expanding colonies, the divisions were predominantly proliferative (88.4% PP, 7.2% PD, and 4.4% DD, Fig. 2A). For divisions observed in the differentiating colonies, terminal differentiation was more frequent (36.2% PP, 31% PD, and 32.8% DD, Fig. 2A). These numbers represent pooled outcomes across all generations and may obscure changes seen over time in vitro.

Thus, we next analyzed the proportions of daughter cell types produced with successive generations. For expanding colonies, the overall proportions of proliferative (P) and differentiated (D) cells produced remained constant at each generation (Fig. 2B), resulting in no detected difference in the proportion of proliferative cells produced over generations (*P* = 0.09, one-way ANOVA). Thus, one population of keratinocytes founds large colonies that maintain near-exponential growth.

When taking keratinocytes from fresh tissue and directly plating them, we are plating keratinocytes that presumably are equally distributed over the stages of differentiation (Fig. 2C). Therefore, to compare cells at similar degrees of differentiation, we analyzed the lineage trees of differentiating colonies working backwards from the terminal differentiation division (TD) to the less differentiated divisions (TD-1, TD-2, etc.). To align expanding colonies to this procedure, we labeled the final generation observed before termination of imaging as final observation (FO).

For differentiating colonies, the proportion of proliferative cells produced (P) declined progressively and the proportion of differentiated cells produced (D) increased from TD-6 to TD-1 (*P* < 0.0001, one-way ANOVA) (Fig. 2D). Thus, a second population of keratinocytes is destined for terminal differentiation and the proportion of D cells increases as differentiation proceeds.

As expected, for expanding colonies, examined backwards (Fig. 2E), similar to that examined forwards (Fig. 2B), the overall proportions of proliferative (P) and differentiated (D) cells produced remained relatively constant over generations (*P* = 0.57, one-way ANOVA).

### Keratinocytes in expanding colonies exhibit a shorter CCD than those in differentiating colonies

**Fig. 3:**
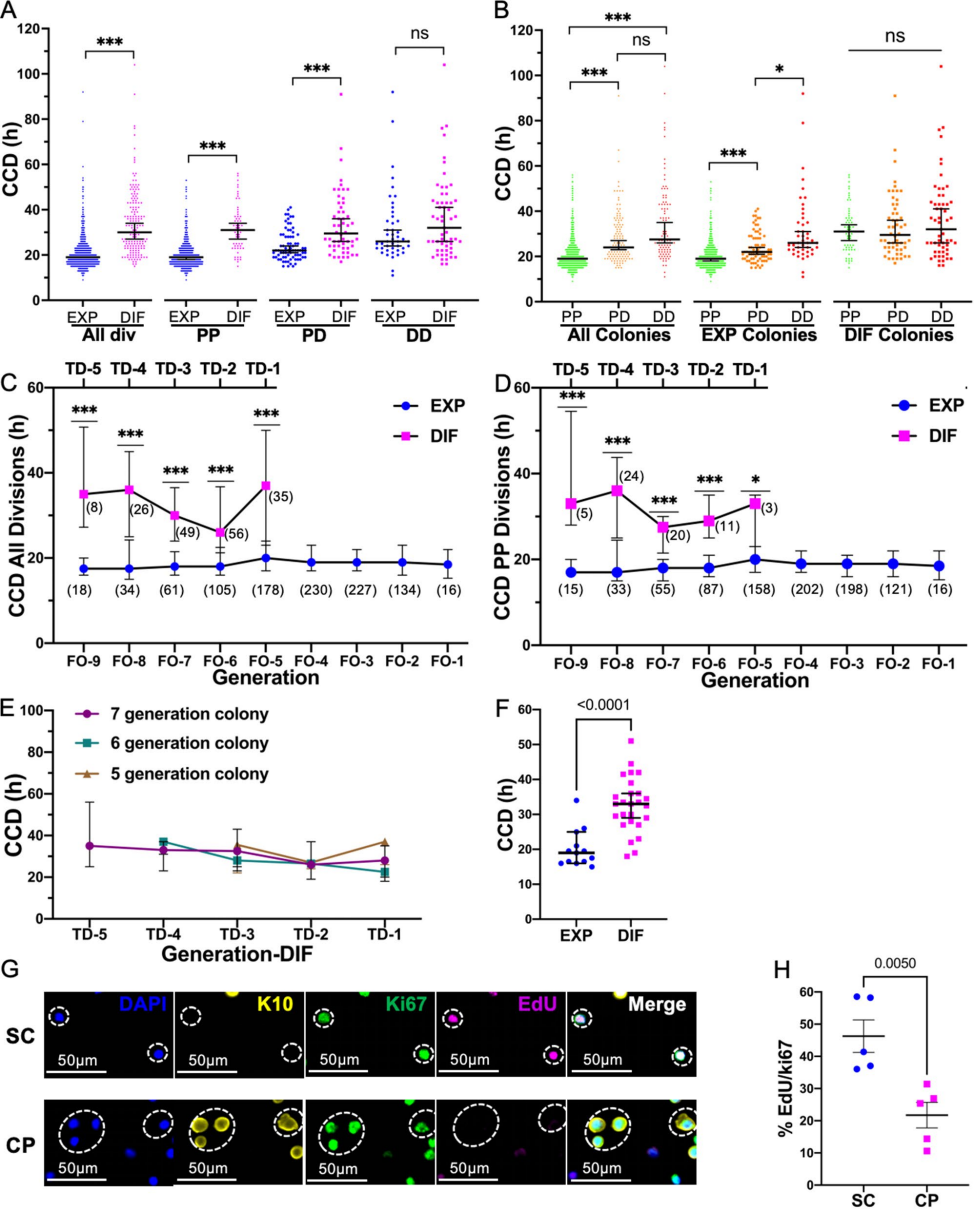
Cell cycle duration analyses of both live cell imaging and EdU incorporation reveal that expanding human keratinocyte colonies have shorter cell cycle durations than differentiating colonies. (**A**) Comparison of CCD of divisions in expanding vs. differentiating colonies. Mann-Whitney test; all divisions: 19 h (*n* = 1017) vs. 30 h (*n* = 174), *P* < 0.0001; PP: 19 h (*n* = 899) vs. 31 h (*n* = 63); PD: 22 h (*n* = 73) vs. 29.5 h (*n* = 54), *P* < 0.0001; DD: 26 h (*n* = 45) vs. 32 h (*n* = 57), *P* = 0.193. (**B**) Comparison of CCD of PP vs. PD vs. DD divisions. Dunn’s multiple comparison test (Kruskal-Wallis post-test). (**C**) CCD of all divisions in expanding vs. differentiating colonies over time. (**D**) CCD of PP divisions in expanding vs. differentiating colonies over time. (**E**) Median CCD for each division/generation (-TD5 to TD-1), for colonies that underwent different numbers of generations before terminal differentiation. (**F**) CCD of all divisions in the first 3 generations of expanding vs. differentiating colonies. Mann-Whitney test, *P* < 0.0001. (**G**) EdU in keratin10^-^Ki67^+^ cycling SCs and early CPs vs. keratin10^+^Ki67^+^ cycling CPs. Scale bar 50 μm. (**H**) Proportion of EdU^+^ (S-phase) cells in cycling CPs vs. SCs and early CPs in (**G**). *t* test, *n* = 5, *P* = 0.005. Data presented as median (95% CI) in A-F or mean ± SEM in H. (): divisions analyzed. EXP: expanding colonies. DIF: differentiating colonies. TD: terminal differentiation. FO: final observation. Not significant (ns) *P* > 0.05. *P < 0.05. ***P < 0.001

During live cell imaging, CCD can be measured as the interval between two consecutive mitoses. Using this method, we found that the median CCD was significantly shorter in expanding vs. differentiating colonies [19 h (*n* = 1017) vs. 30 h (*n* = 174), *P* < 0.0001, Mann-Whitney test] (Fig. 3A). Also, examined individually, the CCDs of PP and PD divisions were each significantly shorter in expanding vs. differentiating colonies [PP: 19 h (*n* = 899) vs. 31 h (*n* = 63), *P* < 0.0001 and PD: 22 h (*n* = 73) vs. 29.5 h (*n* = 54), *P* < 0.0001, Mann-Whitney test], while no significant difference was detected in the CCD of DD divisions [26 h (*n* = 45) vs. 32 h (*n* = 57), *P* = 0.193, Mann-Whitney test]. Thus, in expanding vs. differentiating colonies, the CCD of divisions that produce proliferative cells was shorter, while the CCD of terminal differentiation divisions was similar.

We next compared the CCDs of different division types (Fig. 3B). For expanding colonies, PP, PD, and DD divisions displayed successively longer CCDs (*P* < 0.0001, *P* = 0.04, Dunn’s multiple comparison test, Kruskal-Wallis post-test), while no significant difference was detected in PP, PD, and DD divisions in differentiating colonies (*P* > 0.05, Dunn’s multiple comparison test, Kruskal-Wallis post-test). Thus, proliferative divisions of expanding colonies have uniquely shorter CCDs.

We next examined whether CCD was altered over generations in vitro. Again, as in Fig. 2 we analyzed differentiating colonies backwards from their terminal differentiation (TD), and similarly aligned expanding colonies backwards from the final division observed (FO). No significant difference was detected in the median CCD over generations in culture, in either expanding or differentiated colonies (*P* > 0.05, Kruskal-Wallis, Dunn’s multiple comparison test) (Fig. 3C). Similarly, comparing only PP divisions in either expanding or differentiated colonies, again, no change was detected over generations in culture (*P* > 0.05) (Fig. 3D). However, the CCD of all divisions observed (Fig. 3C) and of PP divisions (Fig. 3D) in expanding colonies was significantly shorter than those in differentiating colonies at each generation (TD-5 to TD-1) (all divisions: *P* < 0.0001 for TD-5 to TD-1. PP divisions: *P* = 0.0007 for TD-5, *P* < 0.0001 for TD-4 to TD-2, *P* = 0.02 for TD-1, Mann-Whitney test). We also included a study of the CCD of divisions in differentiating colonies and expanding colonies, starting with the first division onwards (Additional file 6: Fig. S3).

These studies importantly show that cells in expanding colonies tend to maintain their short CCDs, as a SC phenotype, in culture. Further, and perhaps surprisingly, rather than seeing a progressive lengthening in cell cycle, when a cell is destined to differentiate (CP) it displays a relatively long CCD phenotype, that was not found to change significantly (Fig. 3C-E).

We then focused on the first 3 generations of divisions observed in vitro and found that even within the first 3 generations, the median CCD of all divisions in expanding colonies was significantly shorter than in differentiating colonies [19 h (*n* = 13) vs. 33 h (*n* = 26), *P* < 0.0001, Mann-Whitney test] (Fig. 3F), supporting the possibility of distinguishing expanding vs. differentiating behavior in vitro based on CCD in the early generations.

### Ex vivo, stem cells and early CPs exhibit a shorter cell cycle duration than more differentiated progenitors

Assuming cells in cycle to be CPs or SCs, freshly isolated adult human keratinocytes that were in cycle were identified by expression of Ki67. Expression of Keratin 10 was then used to identify CPs (Keratin 10^+^ Ki67^+^) versus SCs and early CPs (Keratin 10^−^ Ki67^+^). The expression of the S phase marker EdU in these two populations (Fig. 3G, Additional file 5: Fig. S2) revealed 46.3 ± 5.0% S phase SCs and 21.7 ± 3.9% S phase CPs (*n* = 5, *P* = 0.005, *t* test). Applying the ergodicity assumption, this difference in expression indicates that SCs have a shorter CCD than CPs (Fig. 3H). Given the shorter CCD displayed by keratinocytes in expanding colonies, these results support our hypothesis that expanding colonies originate from SCs.

### Single-cell RNA sequencing reveals that the shorter cell cycle seen in stem cells is due to a reduced G1 and G2M

**Fig. 4:**
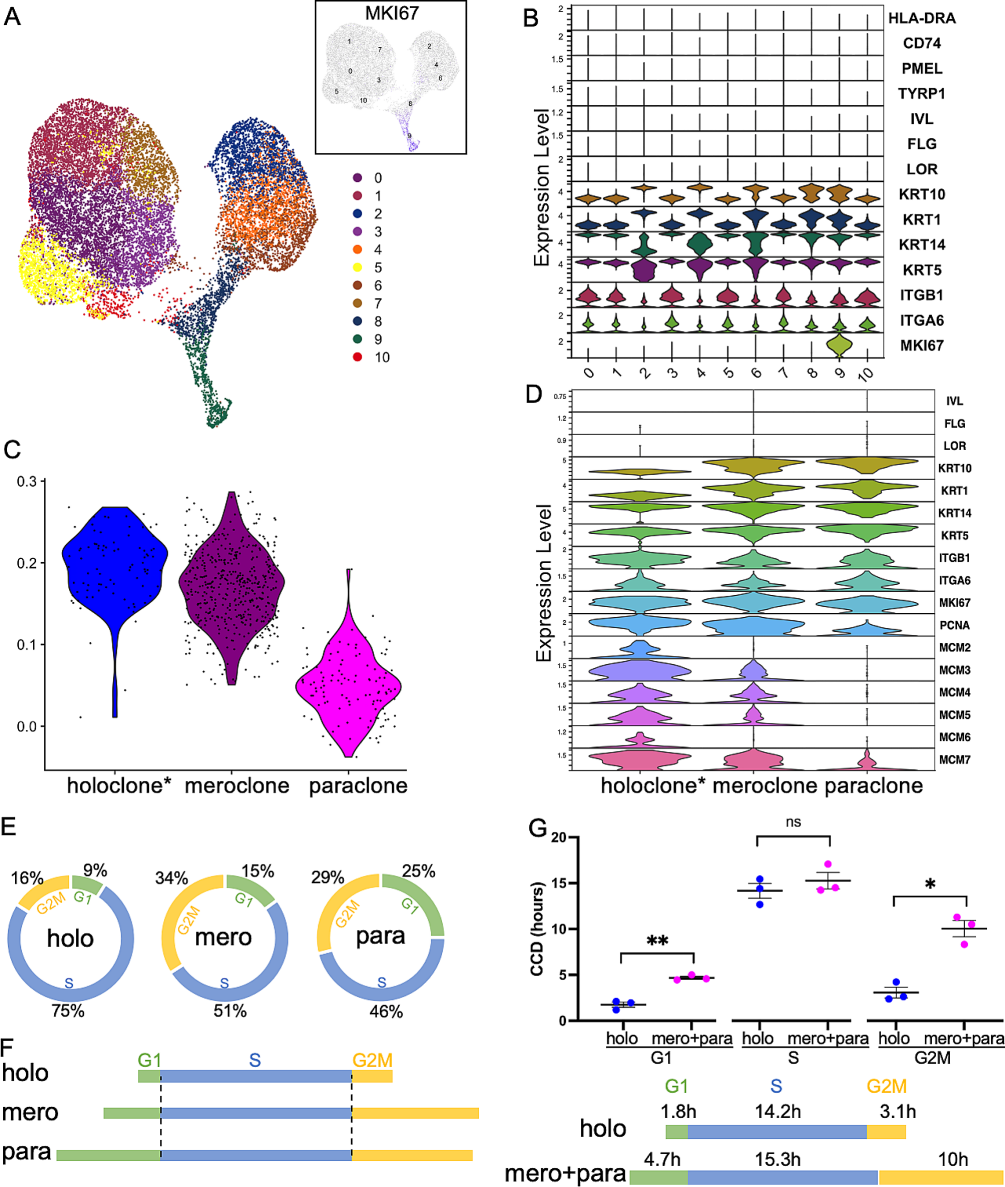
Single cell RNA sequencing shows a decrease in both G1 and G2M phases in human stem cells vs. committed progenitors. (**A**) Umap of *INVOLUCRIN/ LORICRIN/ FILAGGRIN* negative keratinocytes. Cells in cluster 9 express high MKI67. (**B**) Violin plot for populations in (**A**). (**C**) Subclusters of 3 putative progenitor clusters. (**D**) Violin plot for populations in (**C**). (**E**) Cell cycle distribution of holoclones vs. meroclones vs. paraclones using tricycle. (**F**) Inferred differences in cell cycle phase length from (**E**) above, based on the ergodic principle and assumption of equivalent S phase duration. (**G**) Data analysis combining cell phase distribution from Fig. 4E with the median CCDs of EXP vs. DIF colonies from live cell imaging. Data presented as mean ± SEM. Not significant (ns) *P* > 0.05. *P < 0.05. **P < 0.01

To determine the differences in specific cell cycle phases in SCs and CPs, we performed secondary analysis of our previously published single-cell RNA sequencing dataset from normal human adult epidermis (*n* = 3) [30]. Using nonbiased clustering of cells that did not express markers of immune cells, dermal fibroblasts, hematopoietic cells, or differentiated keratinocytes (Involucrin, Filaggrin, and Loricrin) (Fig. 4B), we isolated 22,987 total cells corresponding to basal keratinocytes (Fig. 4B). We focused on the mitotic cluster marked by expression of *MKI67* (Fig. 4A, B), and subclustered 634 total cells into three putative progenitor clusters (Fig. 4C). Recent studies have demonstrated that keratinocyte SCs can be distinguished from CPs utilizing a holoclone transcriptomic signature [28] with relative expression of this signature sequentially reduced in the meroclone and paraclone colonies. We identified the putative holoclone, meroclone and paraclone subclusters by calculating the relative expression of the holoclone signature within the three clusters (Fig. 4D).

We then sought to determine if the cell cycle phase dynamics differ between the three progenitor groups. We utilized a novel cell cycle phase analysis tool, known as tricycle, which has been shown to predict cell cycle phases robustly in several different datasets [31, 32]. Tricycle estimates a cell-cycle embedding by leveraging transfer learning via dimensionality reduction using a fixed reference dataset and projecting new data into this reference embedding, an approach that overcomes key limitations of learning a dataset-dependent embedding. Tricycle then predicts a cell-specific position in the cell cycle based on the data projection [32].

Results of the tricycle analysis showed that more cells were in S phase in the holoclone cluster than in meroclone or paraclone clusters (74.6 ± 4.3% vs. 51.4 ± 3.6% vs. 46.1 ± 2.5%, *P* = 0.003, one way ANOVA), indicating a shorter CCD in the holoclone cluster (Fig. 4E). Using the ergodic assumption and equal S phases, tricycle analysis indicated a decrease in both G1 and G2M in holoclones (SCs) (Fig. 4F). Under the ergodicity assumption, within asynchronous pops, if cell cycle in CPs is longer than that in SCs a smaller proportion of cycling cells will be in S phase. This principle assumes that that cells are evenly distributed around the cell cycle, and that a modification of the length of the cell cycle would be because of changes in the length of G1, not S phase [14–18, 20, 21].

To examine specific phase durations, we combined the cell cycle phase scoring proportions from single cell RNA sequencing (no equal S phase assumption or ergodic principle involved) and the median CCDs seen in live cell imaging. Applying the median CCD of expanding colonies (19 h) to the holoclone cluster, and the median CCD of differentiating colonies (30 h) to the meroclone and paraclone clusters, there was no significant different in the length of S phase (14.2 ± 0.8 h vs. 15.3 ± 0.9 h, *P* = 0.6, paired *t* test), confirming the expected preservation of S phase duration. Shortening of CCD results from the shortening of the G1 (1.8 ± 0.3 h vs. 4.7 ± 0.2 h, paired *t* test, *P* = 0.007) and G2M phases (3.1 ± 0.6 h vs. 10.0 ± 0.9 h, *P* = 0.04, paired *t* test) (Fig. 4G).

Our findings indicate that a short CCD can predict which cells will expand near exponentially in vitro and that the short CCD phenotype can be maintained in culture. The use of EdU and Ki67 expression, confirmed the short CCD as a phenotype of primitive progenitors and SCs. Finally, use of RNA sequencing phase enrichment scores, confirmed an equal S phase duration for human epidermal holoclones vs. meroclones and paraclones, and showed a more than 60% decrease in both G1 and G2M phase durations in holoclones vs. meroclones and paraclones.

## Discussion

These studies examined the range and differences in behavior of adult human keratinocytes over time in culture. Despite the general belief that SCs cycle slowly, studies have found that embryonic and induced pluripotent SCs display shorter CCDs than their more differentiated CPs [9–13]. This study aimed to determine whether the same is true for somatic adult human keratinocyte SCs in cycle.

Live cell imaging revealed two distinct non-overlapping proliferative phenotypes of keratinocytes in culture. Colonies either expanded near-exponentially or terminally differentiated. The keratinocytes in expanding and differentiating colonies had distinct phenotypes of short and long CCDs, respectively. In expanding colonies, the CCD of keratinocytes was significantly lengthened if one of the daughter cells underwent differentiation and was further lengthened if both daughters differentiated. In differentiating colonies, no change was detected in CCD related to division type or generation. Further, examination of newly formed CPs that are plated in live cell imaging studies (the longest-lived early CPs) showed no significant difference in CCD over generations or compared to other CPs (Fig. 3E) indicating not a gradual, but an abrupt shift to a longer CCD in cells committed to differentiation. Thus, overall, there appears to be a distinct lengthening of CCD in keratinocytes destined to differentiate.

We believe that the expanding and differentiating colonies are founded by SCs and CPs, respectively. Indeed, populations of cycling Ki67 + K10- SCs and early CPs displayed a short CCD, as measured by immunofluorescence S phase staining and application of ergodic principles, whereas cycling Ki67 + K10 + CPs displayed a long CCD.

Cell-cycle scoring of single-cell RNA sequencing transcripts from adult human keratinocyte SCs and CPs further indicated a shortened CCD in SCs compared to CPs. This method also indicated that S phase duration was preserved between cell types, while both G1 and G2M were significantly shorter in SCs.

The longitudinal data provided by live cell imaging provided an additional unexpected observation. SCs were able to produce the same proportion of proliferating daughters for up to 14 generations (maximum generations observed), as well as maintain a short CCD in vitro. These findings, along with the holoclone nature of expanding colonies in live cell imaging [7] and the holoclone signature found by Enzo et al. [28], suggest that stemness appears to be preserved during growth in vitro.

Our finding of a dichotomous behavior of adult primary keratinocytes in vitro is in keeping with findings of Nanba et al. and Roshan et al. Reconciliation of the two cell populations seen during live cell imaging studies and the 3 populations observed during secondary cell culture studies of Barrandon and Green was accomplished by Nanba et al. [7]. Using secondary cultures, Nishimura’s group elegantly showed that both meroclones and holoclones are included in the expanding colonies, while stacking/ differentiating colonies are paraclones [7]. Consistent with this, within the expanding colonies we found a small and constant 4.5% of DD divisions that presumably arise from meroclone terminal differentiation (as well as an occasional SC symmetric differentiation).

Our finding of a short CCD phenotype in somatic adult human SCs is similar to findings in embryonic SCs, induced pluripotent SC, and somatic neural SCs. G1 length was found to nearly double during neural SC differentiation, and enabled purification of SCs from heterogeneous cell populations [13]. Further, G1 shortening in neural SCs, by over-expression of cdk4/cyclinD1 inhibited neurogenesis while increasing the generation and expansion of progenitors, and G1 lengthening was necessary and sufficient to switch neural progenitors to neurogenesis [33]. Our single-cell RNA sequencing data similarly showed a more than doubling of G1 duration in keratinocyte CPs. Thus, it is possible that shortening G1 would increase the proliferative capacity of keratinocyte CPs as well.

The colonies that we labeled as differentiating were previously labeled as “balanced”; as they produced approximately equal proportions of P and D daughters, suggesting population asymmetry [29]. We too found that overall, the frequency of PP and DD divisions was similar when all generations are examined together. As a result, the production of P and D daughters was not vastly disparate. However, when similar degrees of differentiation were accounted for, by aligning differentiating colonies from the termianl differentiation division (TD) and working backwards, we found that the likelihood of a DD division increased over the preterminal divisions, from 0 to 20% at F-5 to 100% at terminal differentiation. Thus, while expanding colonies, originating from SCs maintain a constant 88% PP over generations, CPs exhibit a higher likelihood of differentiation divisions at each successive generation.

## Conclusions

The current paradigm is that somatic SCs turn over slowly to protect their DNA from damage, while CPs divide actively to regenerate tissue. In the literature, SCs are repeatedly described as “slow-cycling”. However, frequency of cell division does not equate to rapidity of transit through cell cycle. This work shows that keratinocyte SCs may be more accurately labeled as infrequently but rapidly cycling cells. Future studies may show cell cycle-dependent genes as CP signature markers that can predict proliferative behavior of somatic cells, and manipulation of cell cycle may be used therapeutically to increase proliferation.

### Electronic supplementary material

Below is the link to the electronic supplementary material.

Supplementary Material 1

Supplementary Material 2

Supplementary Material 3

Supplementary Material 4

Supplementary Material 5

Supplementary Material 6

## Data Availability

Detailed procedures related to isolation of human keratinocytes, live cell imaging, lineage analysis, immunostaining/ S phase labeling, single-cell RNA sequencing and cell cycle phase analysis, and statistical analysis, as described in Materials and Methods, will be shared by the lead contact upon request. RNA-seq data EGAS00001002927 were submitted to European Genome-phenome Archive (EGA) database (https://ega-archive.org/studies/EGAS00001002927). This paper does not report original codes.

## Abbreviations

SC: Stem cell
CP: Committed progenitor
CCD: Cell cycle duration
