# Supplementary figures and images for: Short cell cycle duration is a phenotype of human epidermal stem cells
Source: Stem Cell Res Ther. 2024 Mar 13;15:76. doi: 10.1186/s13287-024-03670-y (PMC10935907; doi:10.1186/s13287-024-03670-y)

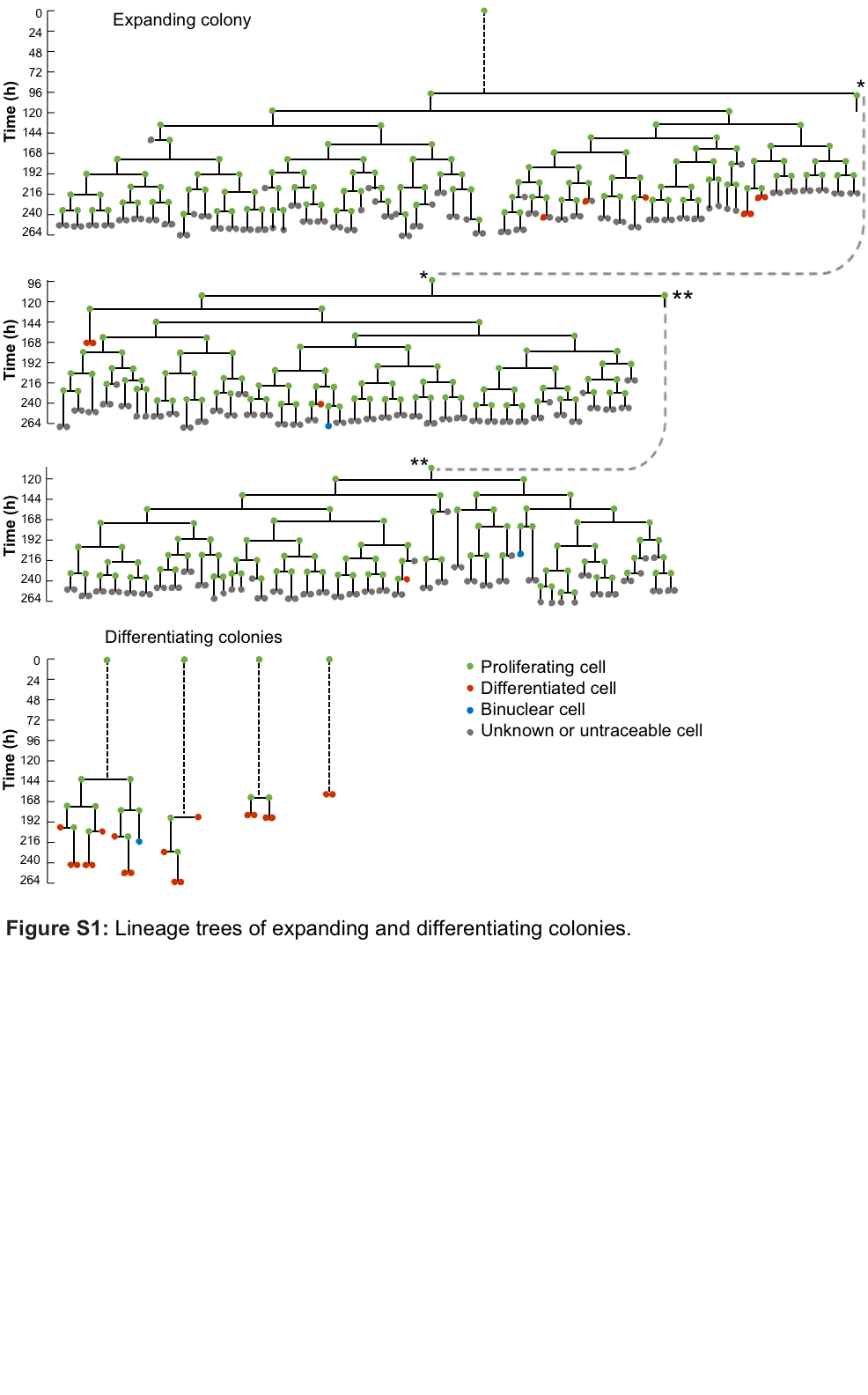

Supplement: Supplementary file 4 — Supplementary Material 4 [file 13287_2024_3670_MOESM4_ESM.tif]

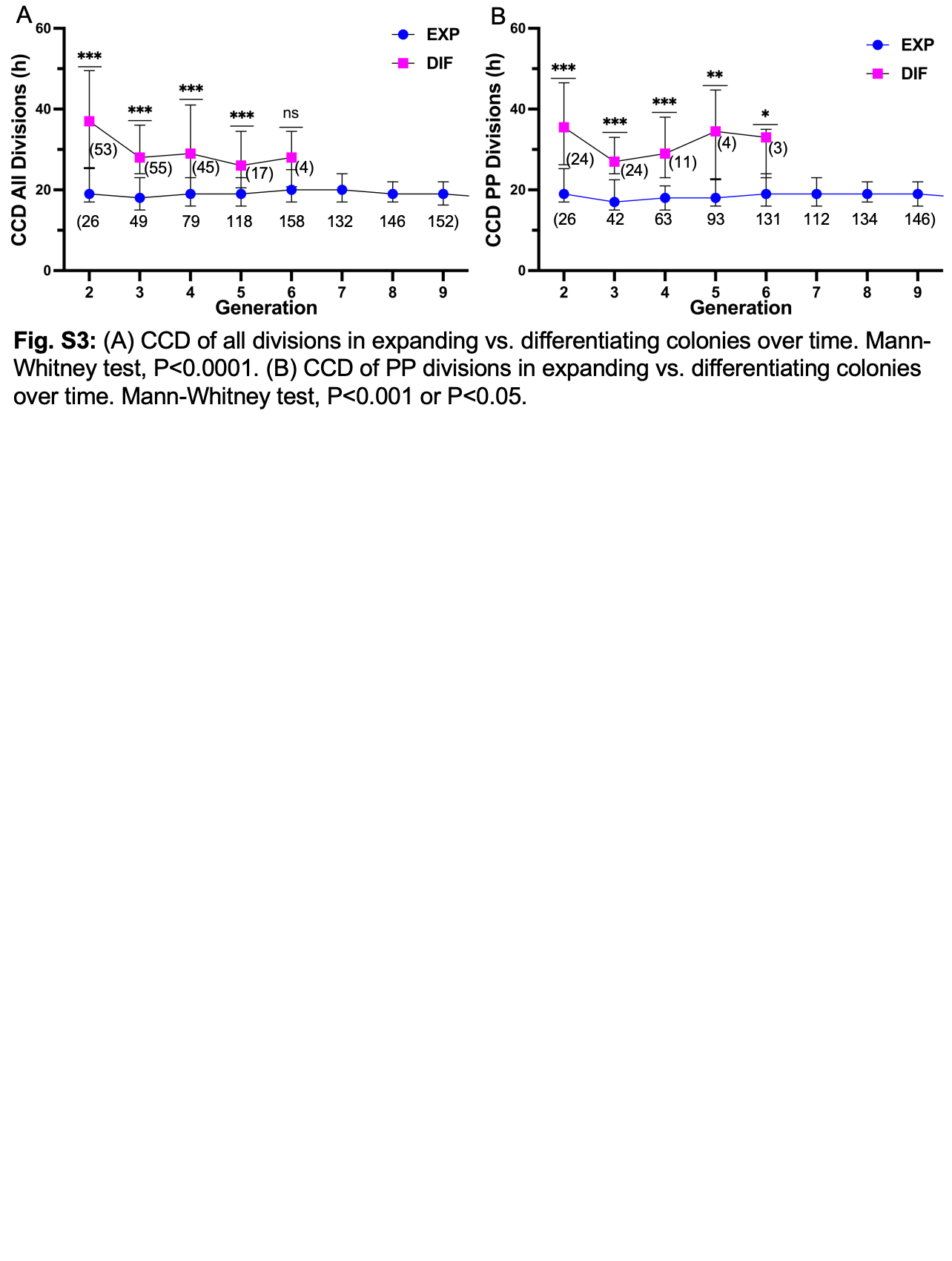

Supplement: Supplementary file 5 — Supplementary Material 5 [file 13287_2024_3670_MOESM5_ESM.tif]

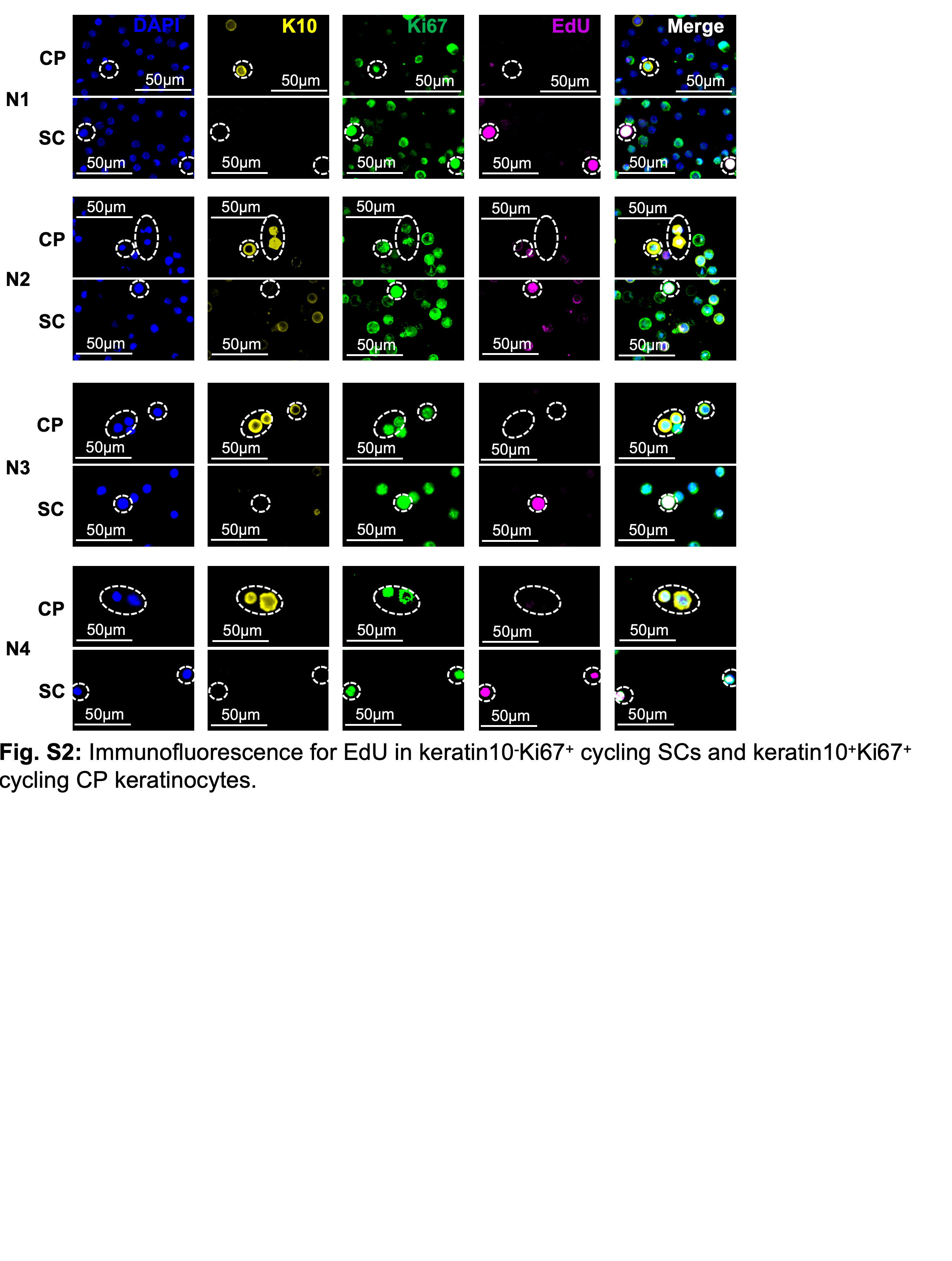

Supplement: Supplementary file 6 — Supplementary Material 6 [file 13287_2024_3670_MOESM6_ESM.tif]
